# Macronutrient intake and simulated infection threat independently affect life history traits of male decorated crickets

**DOI:** 10.1002/ece3.6813

**Published:** 2020-09-22

**Authors:** Kristin R. Duffield, Kylie J. Hampton, Thomas M. Houslay, James Rapkin, John Hunt, Ben M. Sadd, Scott K. Sakaluk

**Affiliations:** ^1^ Behavior, Ecology, Evolution and Systematics Section School of Biological Sciences Illinois State University Normal IL USA; ^2^ Department of Zoology University of Cambridge Cambridge UK; ^3^ Centre for Ecology and Conservation University of Exeter Penryn UK; ^4^ School of Science and Hawkesbury Institute for the Environment Western Sydney University Penrith NSW Australia; ^5^ Present address: Crop Bioprotection Research Unit United States Department of Agriculture National Center for Agricultural Utilization Research, Agricultural Research Service Peoria IL USA

**Keywords:** cricket, diet, immunity, life history theory, reproductive effort, terminal investment

## Abstract

Nutritional geometry has advanced our understanding of how macronutrients (e.g., proteins and carbohydrates) influence the expression of life history traits and their corresponding trade‐offs. For example, recent work has revealed that reproduction and immune function in male decorated crickets are optimized at very different protein:carbohydrate (P:C) dietary ratios. However, it is unclear how an individual's macronutrient intake interacts with its perceived infection status to determine investment in reproduction or other key life history traits. Here, we employed a fully factorial design in which calling effort and immune function were quantified for male crickets fed either diets previously demonstrated to maximize calling effort (P:C = 1:8) or immune function (P:C = 5:1), and then administered a treatment from a spectrum of increasing infection cue intensity using heat‐killed bacteria. Both diet and a simulated infection threat independently influenced the survival, immunity, and reproductive effort of males. If they called, males increased calling effort at the low infection cue dose, consistent with the terminal investment hypothesis, but interpretation of responses at the higher threat levels was hampered by the differential mortality of males across infection cue and diet treatments. A high protein, low carbohydrate diet severely reduced the health, survival, and overall fitness of male crickets. There was, however, no evidence of an interaction between diet and infection cue dose on calling effort, suggesting that the threshold for terminal investment was not contingent on diet as investigated here.

## Graphical abstract

Reproduction and immune function in male decorated crickets are optimized at very different macronutrient dietary ratios. However, it is unclear how an individual's diet interacts with its perceived infection status to determine investment in reproduction or other key life history traits. Here, we found that macronutrient intake and a simulated infection threat influence the expression of life history traits in decorated crickets, but do not interact, suggesting that terminal investment is not contingent on diet.

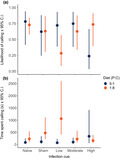

## 1 INTRODUCTION

Resource availability and acquisition are central to life history investment (Gadgil & Bossert, 1970; Roff, 1992; Stearns, 1992; Williams, 1966). Due to competition for resources, negative associations (i.e., physiological trade‐offs) between traits often arise when organisms are resource‐limited (Gadgil & Bossert, 1970; van Noordwijk & de Jong, 1986; Perrin & Sibly, 1993; Zera & Harshman, 2001; Zera et al., 1998). Allocation of resources to reproduction versus survival, including immune function, represents one such key physiological trade‐off (Lochmiller & Deerenberg, 2000; Martin et al., 2008; McKean et al., 2008; Norris & Evans, 2000; Partridge et al., 2005; Schwenke et al., 2016; Sheldon & Verhulst, 1996), and numerous negative associations between these traits have been reported in food‐limited organisms including nematodes (Klass, 1977), insects (Chapman et al., 1994; Chippindale et al., 1993; Tatar & Carey, 1995), birds (Ardia, 2005; Bonneaud et al., 2004; Gustafsson et al., 1994; Ilmonen et al., 2000), lizards (Cox et al., 2010; French et al., 2007), and mammals (Koivula et al., 2003).

Beyond the consequences for trade‐offs dictated by the number of total calories consumed, only recently have investigators begun to consider the independent effects of macronutrients (i.e., carbohydrates, protein, and fat) on an individual's life history strategy. This was largely driven by the development of nutritional geometry (Simpson et al., 2017; Simpson & Raubenheimer, 1995), a multidimensional framework for disentangling the effects of energy consumption from those of particular nutrient combinations. The application of this framework has revealed that the ratios of macronutrients consumed often mediate life history trade‐offs, including those between lifespan and reproduction (Jensen et al., 2015; Lee et al., 2008; Maklakov et al., 2008; Rapkin, et al., 2017; Solon‐Biet et al., 2015) and growth and immune function (Cotter et al., 2011).

A recent application of nutritional geometry has illuminated the nutritional landscape underlying the trade‐off between immune function (i.e., mounting an encapsulation response) and reproductive effort (i.e., time spent broadcasting a long‐range acoustic call for mate attraction) in male crickets, *Gryllodes sigillatus* (Rapkin et al., 2018). As in other crickets, male *G. sigillatus* must call in order to attract receptive females to mate, and so it follows that calling effort is an integral aspect of reproductive effort in this species. In this study, crickets were maintained on one of 24 holidic diets varying in protein:carbohydrate (P:C) ratio and total nutritional content that yielded a nutritional landscape with six nutritional rails, along which P:C ratio was held constant but total calories differed. Nutrient intake was measured during sexual maturation, and calling effort and encapsulation ability were subsequently quantified. Rapkin et al. (2018) found that immune function in males was maximized at a P:C ratio of approximately 5:1, whereas calling effort was maximized at a carbohydrate‐biased P:C ratio of approximately 1:8. Because optimal expression of each trait occurred at distinct P:C ratios, this study provides important evidence that macronutrient intake, independent of total calories, directs the trade‐off between reproduction and immune function.

The ratio of macronutrients available to an individual and the influence on reproduction and immunity may alter the life history investment strategy employed following a pathogenic infection. On one hand, the cost of immunity trade‐off hypothesis predicts an increased investment in immunity to fight infection, at a cost to current reproduction (Adamo, 1999; Ahtiainen et al., 2005; Festa‐Bianchet, 1988; Gustafsson et al., 1994; Jacot et al., 2004; Norris et al., 1994; Stahlschmidt et al., 2013; Svensson et al., 1998). Alternatively, individuals may increase their investment in reproduction in response to the threat of infection to their immediate survival, in a life history strategy known as terminal investment (Clutton‐Brock, 1984; Williams, 1966). A recent refinement of this idea, the dynamic terminal investment threshold model, proposes that the tendency of an individual to terminally invest depends on other intrinsic and extrinsic factors, such as age or diet, that alter an individual's residual reproductive value (i.e., expectation for future offspring) (Duffield et al., 2017). In support of this possibility, Hudson et al. (2019) recently demonstrated that the propensity to terminally invest in female *Drosophila melanogaster* was contingent on the amount of protein consumed. Specifically, female flies only expressed terminal investment (i.e., increased egg laying following an infection of *Pseudomonas aeruginosa*) when they were fed a high protein diet, whereas infected females fed a diet of lower total protein did not increase egg production relative to uninfected females. This study highlights how nutrition‐dependent condition, based on the amount of protein consumed in this example, may interact with infection status to determine an individual's investment in key life history traits, including reproduction.

Here, we explore how macronutrient intake interacts with simulated infection cue intensity, achieved through a spectrum of immune challenge treatments, to influence expression of life history traits in male decorated crickets, *G. sigillatus*. Specifically, we quantified reproductive effort (calling effort) and immune function (circulating hemocytes, presence of hemocyte microaggregations, and humoral antibacterial activity of the hemolymph) of males maintained on diets previously shown to maximize calling effort (P:C = 1:8, “high carbohydrate diet”) or a component of immune function, specifically encapsulation ability (P:C = 5:1, “high protein diet”) (Rapkin et al., 2018). Due to the divergent nutritional demands of calling effort and immune function, we predicted that males would exhibit significantly different life history strategies depending on their macronutrient intake, infection cue intensity, or the interaction between these factors. We particularly predicted that males maintained on a diet that maximizes calling effort would terminally invest in calling effort at a lower infection cue dose than males maintained on a diet that maximizes immune function.

## 2 MATERIALS AND METHODS

### 2.1 Study animals

Male *G. sigillatus* used in this study were randomly selected from a large outbred colony, initiated with crickets collected from a wild population from Riverside, CA in 2014. All crickets used in this study were of the same generation. Newly hatched, experimental crickets were reared in 55 L plastic storage bins packed with egg carton to increase rearing surface area and provisioned with a standard diet (roughly equal parts Envigo^©^ 2018 CM Teklad Certified Global 18% protein rodent diet and Purina^®^ Cat Chow Complete pellets) and water (glass vials plugged with moist cotton) ad libitum. The nutritional content for this standard diet is 18.6% protein, 6.2% fat, 44.2% carbohydrate (P:C = 1:2.4) for the rodent diet, 32.0% protein, 12.0% fat, and 37.6% carbohydrate (P:C = 1:1.2) for the Purina® Cat Chow. When sex differences became apparent (4th or 5th instar), juvenile males were removed from stock colonies and housed individually in small (450 ml) plastic containers, ensuring virgin status and age control for all experimental crickets, and provisioned with food (roughly equal parts rodent diet and cat food) and water (glass vials plugged with cotton) ad libitum. A small section of egg carton was also provided as a refuge. All individuals were housed in an environmental chamber at 32°C on a 16 hr:8 hr light:dark cycle. Males were checked daily to determine the date of final molt to adulthood and then randomly assigned to a diet treatment.

### 2.2 Artificial diets and measuring dietary intake

Isocaloric diets (which differed in P:C but not total calories) were created using a protocol from an earlier study that quantified the amount of carbohydrates and protein maximizing calling effort (P:C 1:8) and immune function (i.e., encapsulation ability) (P:C 5:1) in male *G. sigillatus* (Rapkin et al., 2018). Briefly, proteins consisted of a 3:1:1 mixture of casein, albumen, and peptone, with digestible carbohydrates consisting of a 1∶1 mixture of sucrose and dextrin. All diets contained Wesson's salts (2.5%), ascorbic acid (0.28%), cholesterol (0.55%), and a vitamin mix (0.18%). After the appropriate dry weight of protein and carbohydrate had been added to the mixture, the remainder of the mixture was adjusted to the appropriate composition with crystalline cellulose.

Upon adult eclosion, each cricket was given one dish of his assigned diet (high protein diet: *n* = 249; high carbohydrate diet: *n* = 245), which was changed every 3 days over a period of 21 days (7 trays total for each male). Diet was provided in feeding platforms created by gluing the upturned plastic lid of a 2‐ml microcentrifuge tube in the center of a plastic petri dish (50 mm diameter, 9 mm deep). The materials and design of the feeding platforms ensured that food could be collected in the petri dish if any spilled while subjects were feeding. Diet consumption was calculated as the difference in dry weight before and after feeding. Prior to weighing, any feces were removed from the feeding platform using fine forceps. The dry weight of all food‐containing trays, obtained by holding them in drying oven at 30°C for at least 72 hr, was measured both before and after feeding to quantify how much of each diet was consumed by an individual throughout the feeding trials, as this alters the total amount of nutrients available. This is an especially important facet of evaluating the effects of macronutrients, but one not often considered in such assessments.

### 2.3 Infection cue

A pathogenic infection may signal reduced residual reproductive value to the host, and this may be mediated through the infection‐associated immune challenge. Earlier, we demonstrated that injection with heat‐killed *Escherichia coli* stimulates an immune response and invokes a terminal investment response in male *G. sigillatus* (Duffield et al., 2015, 2018, 2019). This approach ensures that the measured response is related to the host's investment strategy, and not the result of alternative causes related to a live infection; thus, a similar protocol was implemented here. Males were given an infection cue treatment following the 3‐week feeding trials based on a previous study demonstrating that males of this age are more likely to show terminal investment in calling (Duffield et al., 2018). Body mass for each male was measured immediately prior to infection cue administration using an analytical balance (Mettler Toledo AG245).

Males were randomly assigned to one of five treatments on an increasing infection cue spectrum: (a) naive (unmanipulated control), (b) sham control (injection of 2 μl ringer saline), (c) low‐dose infection cue (injection of 2 μl ringer saline with 5 × 10^5^/ml heat‐killed *E. coli*), (d) moderate‐dose infection cue (injection of 2 μl ringer saline with 5 × 10^7^/ml heat‐killed *E. coli*), or (e) high‐dose infection cue (injection of 2 μl ringer saline with 5 × 10^8^/ml heat‐killed *E. coli*). Importantly, we consider the sham control treatment not only a control for the effect of injection, but also as a low‐level mortality threat because injection causes cuticle damage and induces an immune response that potentially signals a mortality threat to the individual (Ardia et al., 2012; Gillespie & Khachatourians, 1992; Wigby et al., 2008). Injections were performed using a 5 µl syringe with a 1 mm compression fitting (Hamilton^®^ brand) within which a needle formed from a heat‐pulled glass capillary tube was inserted. Crickets were injected between the 6th and 7th pleurite of the thorax. Pulled capillaries were cleaned in 70% ethanol, rinsed with ultrapure water, and dried between each injection, and capillaries were not reused across treatments or days. Treatments were always applied at the same time (0900 hr ± 1 hr) throughout the experiment. Owing to mortality during the 3‐week feeding period, our sample sizes for each treatment were *high protein diet*: naive = 33, sham = 37, low = 35, moderate = 34, high = 42; *high carbohydrate diet*: naive = 40, sham = 41, low = 41, moderate = 43, high = 47.

*Escherichia coli* (ATCC strain 23716) used to create our infection cues were cultured at 30°C in 7 ml of liquid medium (10 g bacto‐tryptone, 5 g yeast extract, 10 g NaCl in 1,000 ml of distilled water, pH 7). To prepare bacterial suspensions for immune challenge injections, 1 ml of an overnight culture was centrifuged (850 *g*, 4°C, 10 min), the supernatant discarded and replaced with sterile ringer saline. This procedure was repeated three times. The bacteria were then heat‐killed (90°C, 5 min), and the concentration of bacterial cells was adjusted to the concentrations described earlier for each infection cue dose. Efficacy of the heat‐killing was confirmed by plating out samples of the suspension on media agar.

### 2.4 Assessing reproductive effort

Calling effort of males was used as a proxy for reproductive effort. Male *G. sigillatus* begin mating 4.5 days on average after adult eclosion (Burpee & Sakaluk, 1993) and, because calling is essential for mating, it follows that males are also calling at this time. We quantified calling effort (i.e., the amount of time males spent calling) over two consecutive nights following infection cue treatment. Calling effort was measured using a custom‐built high‐throughput sound monitoring array (Bertram & Johnson, 1998; Duffield et al., 2018, 2019) in which each male‐containing individual container (250 ml) was fitted with a lid‐mounted microphone (C1163, Dick Smith Electronics) and placed within a small Styrofoam box to prevent crosstalk between containers. Following administration of infection cues, males were provisioned with water and a small piece of egg carton for refuge and given 7 hr (± 1 hr) to acclimate prior to the start of recording trials. Males did not have access to food during the recording period to prevent any false positives caused by audible food consumption. Recording periods started at 1,700 hr and ran for 39 hr, ending at 0800 hr 2 days following treatment; this period captures calling effort at times most relevant to female mate attraction (Burpee & Sakaluk, 1993; Sakaluk, 1987; Sakaluk et al., 2002). The sound monitor sampled each microphone throughout the recording periods every 2 s, and based on the binary output resulting from this protocol, total calling time was calculated for each male across recording periods (Duffield et al., 2018, 2019; Hunt et al., 2004).

### 2.5 Survival and postmortem measurements

Following the end of the two‐night calling trials, males were returned to their original individual rearing boxes and provided standard diet and water ad libitum. Mortality was monitored and recorded daily. Upon death, pronotum width was measured as a proxy for structural body size, using a stereomicroscope (Nikon SMZ800) equipped with a digital camera and imaging software (Nikon NIS‐Elements Documentation v. 4.20).

### 2.6 Quantifying immune function

We measured multiple immune parameters to capture a suite of immune pathways that may differ between diets or be altered following infection cue treatment. Here, we analyzed antibacterial activity, total circulating hemocytes, and the presence of hemocyte microaggregations from hemolymph that was collected immediately after calling effort trials. General cell‐free antimicrobial activity of hemolymph is a component of the humoral immune response of insects and includes the action of both lysozyme‐like enzymes and antimicrobial peptides (Lemaitre et al., 1997). Additionally, hemocytes are a component of the cellular response of insect immunity and are involved in core processes that include coagulation, nodule formation, phagocytosis, and encapsulation (Lavine & Strand, 2002). Finally, the microaggregation of hemocytes is an early step in the process of nodule formation (Miller et al., 1999; Miller & Stanley, 2004), a key insect cellular defense reaction to bacterial challenges and responsible for clearing a large proportion of bacteria from circulation (Howard et al., 1998). To collect hemolymph, males were cold‐anesthetized, and the membrane was pierced under the anterior of the dorsal pronotum plate with a sterile 25 G needle, and 5 µl of outflowing hemolymph taken with a prechilled glass microcapillary tube positioned at the puncture site. Collected hemolymph was then expelled into 11 µl of chilled Grace's insect medium (MilliporeSigma) to be used in antibacterial assays; 5 µl of this mixture was then added to 15 µl of chilled Grace's insect medium to be immediately used for circulating hemocyte counts and determining the presence of microaggregations. The samples for antibacterial assays were snap‐frozen in liquid nitrogen and stored at −80°C for later analysis.

#### 2.6.1 Zone of inhibition assay

Although immune‐challenged individuals in this study were injected with *E. coli*, previous assays resulted in no measurable antibacterial activity on plates seeded with *E. coli*. Therefore, antimicrobial activity was assayed from zones of inhibition induced by samples on petri dishes containing agar seeded with *Micrococcus luteus* (ATCC 4698) (see Sadd and Schmid‐Hempel, 2007 for methodological details). Briefly, *M. luteus* from a single colony on a streak plate were incubated in a shaking incubator overnight at 30°C in 7 ml of media (2.5 g peptone and 1.5 g meat extract in 500 ml of nanopure water, pH 7.0). From this culture, bacteria were added to liquid media containing 1% agar held at 40°C to achieve a final density of 1.5 × 10^5^ bacterial cells/ml. Six milliliters of seeded medium were added to a 100 mm diameter petri dish to solidify. Sample wells were made using a Pasteur pipette (Volac D810) fitted with a ball pump, and 2 µl of sample hemolymph solution thawed on ice was added to each well. Negative (Grace's insect medium, Thermo Fisher Scientific, CAS: 11605094) control wells were also included on each plate. Plates were inverted, incubated for 48 hr at 30°C, and then the diameter of inhibition zones was measured for each sample. Two diameter measurements of zones, perpendicular to one another, were measured for each sample using ImageJ (Schneider et al., 2012) and averaged (measurements were performed blind to treatment). Because zone of inhibition diameter does not increase linearly with antibacterial activity, measured zone diameters were converted, based on a standard curve, to units (mg/mL) of lysozyme (from hen egg white, MilliporeSigma, CAS: 12650‐88‐3). Each hemolymph sample was tested in duplicate, with the mean of the duplicates being used in analyses.

#### 2.6.2 Circulating hemocyte and microaggregation counts

Hemocytes were counted, and microaggregations were observed at 400× magnification under a phase‐contrast microscope in Fast‐Read 102^®^ plastic counting chambers to assess their numbers per individual as a proxy for cellular immunity (Duffield et al., 2018; King & Hillyer, 2013; Stoepler et al., 2013). Counting was performed blind to diet and infection cue treatment.

### 2.7 Statistical analyses

All statistical analyses were done in R (version 3.6.1 or 3.6.2, R Core Team, 2019). Reported results derive from the best models as determined by corrected Akaike's information criterion (AICc using the stepAIC function in R; Hurvich & Tsai, 1989; Sugiura, 1978) or before the removal of terms from the final model.

#### 2.7.1 Amount of diet consumed

To assess the amount of diet consumed during the feeding trials, we ran a linear mixed model using the lme4 package (Bates et al., 2015), including pronotum width (a proxy for structural body size), diet (high protein or high carbohydrate), and tray number (1–7) as fixed effects, including all associated interactions, and individual identification as a random effect. We calculated the average amount of food consumed for each male by adding the total dry weight of food consumed and dividing by the number of trays measured. To analyze the average amount of food eaten, we included the main effect of diet, pronotum width as a covariate, and the interaction between the two in a generalized linear model with a Gamma error distribution and log link.

#### 2.7.2 Reproductive effort

Given the large number of very short calls in our data set and the susceptibility of our sound monitor system to false positives to outside noise or sudden movements of crickets within boxes, we followed standard procedure and removed 5 s from each measurement and then rounded any negative measurements to zero (see also Duffield et al., 2018, 2019). The final distribution of male calls indicated that calling effort measurements were zero‐inflated and overdispersed, and so we analyzed the data using a zero‐altered Poisson (ZAP) model fitted with the package MCMCglmm (Hadfield, 2010). The ZAP model includes both a logistic regression for the zero/nonzero component of the data (i.e., identifying which factors affect whether a male calls or not) and an overdispersed Poisson regression for the zero‐truncated counts (identifying which factors affect the amount of calling given that a male calls).

We used binary indicator variables (0/1) to specify whether or not a male belonged to each infection cue group and diet (Gelman & Hill, 2007). The reference group for our model was the naive infection cue and the carbohydrate‐biased (P:C 1:8) diet. Our predictors then included the indicator for high protein (P:C 5:1) diet, 4 indicators for infection cue, the interaction between diet and each infection cue, and also the average amount of food eaten by that individual (centered at the mean and scaled to standard deviation units). We ran the model for 550,000 iterations, with a burn‐in of 50,000 and retaining every 100th sample. Fixed effects are considered statistically significant if the 95% highest posterior density credible intervals exclude 0. For model diagnostics, we used visual checks of the thinned chains and ran the model 3 times to ensure convergence to a similar posterior distribution (Gelman & Rubin, 1992). The model used an uninformative prior for the Poisson regression and fixed the residual variance to 1 for the logistic regression. To check that the posterior distributions were not heavily influenced by the prior distributions, we ran the models with multiple different prior specifications.

#### 2.7.3 Immune measures

The full models for all immune measures (circulating hemocytes, presence of circulating microaggregations, and zones of antibacterial activity) included diet type, infection cue, and the interaction between the two (main effects) as well as the average amount of diet eaten and pronotum width (covariates). Circulating hemocytes were analyzed with a negative binomial generalized linear model, the presence of circulating microaggregations of hemocytes was analyzed using a generalized linear model with a binomial distribution, and the zones of antibacterial activity were analyzed with a generalized linear model with a Gamma error distribution.

#### 2.7.4 Mortality during and survival following diet treatments

To investigate the effects of diet and infection cue treatments on survival throughout the experiment, we assessed mortality and survival at three points: mortality during the 3‐week feeding period, mortality during the 2‐day calling trial, and survival following calling effort trials. To assess mortality during both feeding trials, the full model included diet type, the average amount of food eaten, and the interaction between the two as main effects and pronotum size as a covariate. The full models for mortality during the calling trial and survival following included diet type, infection cue, and the interaction between the two as main effects and average amount of diet eaten and pronotum width as covariates. Mortality during both feeding trials and calling effort trials was assessed using a generalized linear model with a quasibinomial distribution (to accommodate overdispersion of the data), and survival following calling trials was analyzed using a Cox proportional hazards regression model.

## 3 RESULTS

### 3.1 Amount of diet consumed

**FIGURE 1:**
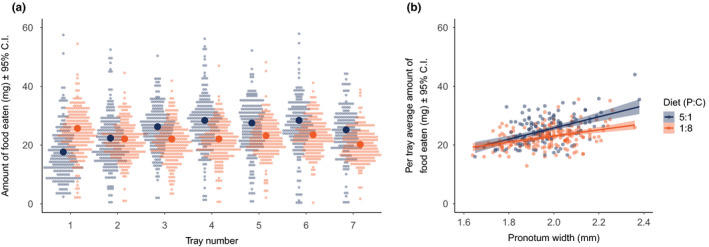
Diet consumption by male *Gryllodes sigillatus* fed either of two diet types (P:C = 5:1 and 1:8). (a) The amount of food eaten (dry weight of trays containing diet both before and after offering) across tray number (i.e., feeding period time). (b) The average amount of diet eaten during the feeding trial between diet types and based on pronotum width, a proxy of structural body size. Feeding periods were 3 weeks long, and trays were switched every 3 days for each male

We found a significant three‐way interaction between diet, pronotum width, and tray number (i.e., time period of feeding trial) on the amount of food that males consumed during the 3‐week feeding period (*F*_6, 1776.32_ = 3.16; *p* = .0044; Figure 1). Males held on the high carbohydrate diet (P:C 1:8) consumed more food than those maintained on the high protein diet (P:C 5:1) early in the feeding period, but after the second tray, this pattern was reversed, and males on the high protein diet (P:C 5:1) consistently ate more food than those on the high carbohydrate diet (Figure 1a). There was also a significant interaction between diet and pronotum width (ANOVA: *F*_1, 313_ = 3.89; *p* = .04954) with respect to the average amount of food eaten, which increased with body size in both treatments, but more steeply in males held on the high protein diet (Figure 1b). To account for this size‐based variation in food consumed in subsequent analyses, we included the average amount of food consumed for each individual, as well as their pronotum width, as covariates in the full models.

### 3.2 Mortality

**FIGURE 2:**
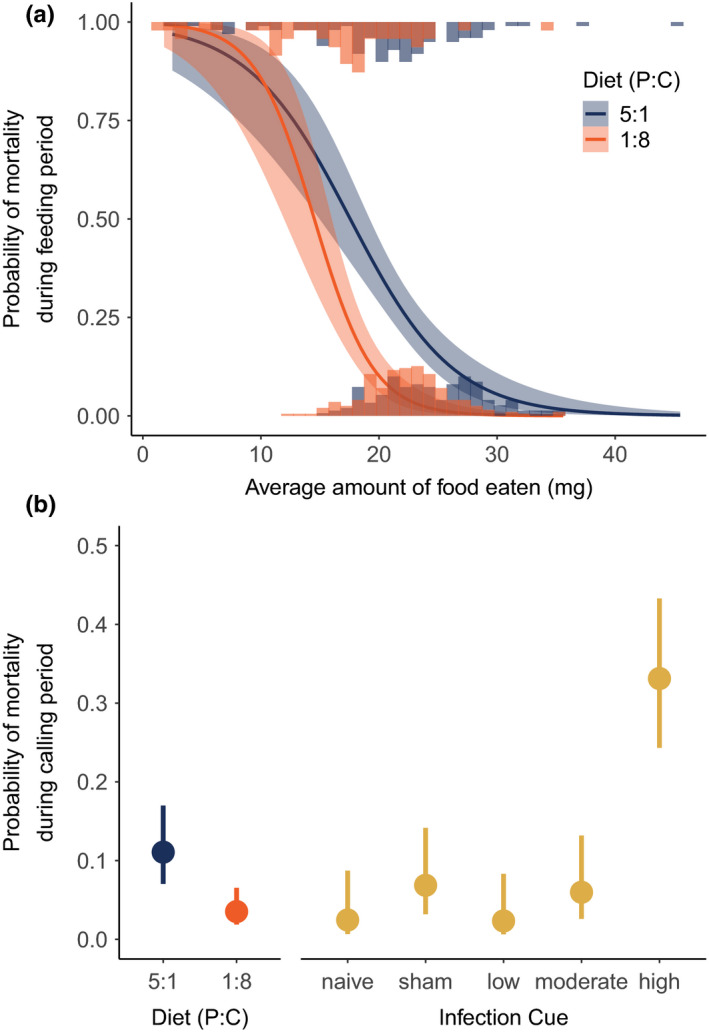
The probability of mortality of male *Gryllodes sigillatus* during: (a) the 3‐week feeding trial, based on diet and amount of food eaten, and (b) the subsequent 2‐day calling period based on diet and infection cue treatments

We found considerable diet‐dependent mortality during the experiment. Specifically, there was a significant interaction between the average amount of food eaten and diet type in their effect on mortality during the 3‐week feeding period (*F*_1,488_ = 4.8509; *p* = .0281). Mortality decreased with average mass of food eaten in both treatments, but mortality was higher in males held on the high protein diet, at all levels of food consumed, except at lower consumption (Figure 2a). Additionally, both diet (*F*_1,387_ = 13.514; *p* < .001) and infection cue (*F*_4,387_ = 14.427; *p* < .001) significantly affected survival through the 2‐day calling trials; in this case, the interaction term was not significant (*F*_4,382_ = 1.032; *p* = .3905) and was not included in the final model. Males held on the high protein diet were the least likely to survive through the end of the 2‐day calling trial. Controlling for diet, males receiving the highest dose infection cue also were the least likely to survive the 2‐day calling trial (Figure 2b). Males that died prior to the conclusion of the calling trials were eliminated from further analyses of reproductive effort, immune function, or survival.

### 3.3 Reproductive effort

**FIGURE 3:**
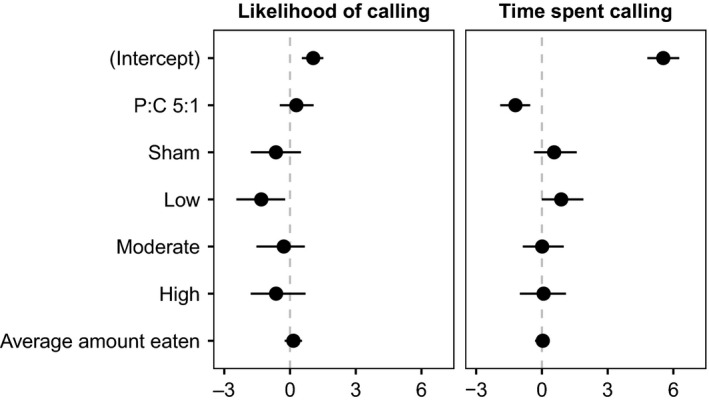
Model coefficients with 95% confidence intervals from the MCMCglmm zero‐altered Poisson (ZAP) analysis of the likelihood of calling (a logistic regression for zeros) and time spent calling (zero‐truncated Poisson) in male crickets (*Gryllodes sigillatus*) fed either of two diet types and administered an infection cue from a gradient of increasing intensity using heat‐killed bacteria. Diet effects are shown as the deviation from the P:C 1:8 diet treatment, and overall infection effects are shown as differences from the naive level

**FIGURE 4:**
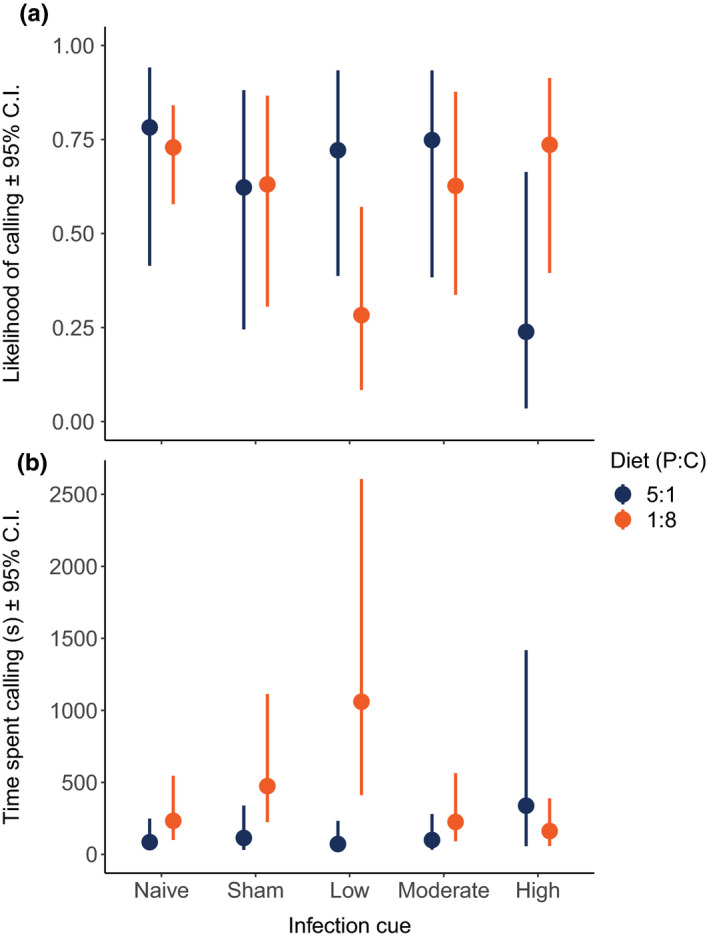
Model predicted effects of diet type and infection cue (from a gradient of increasing intensity using heat‐killed bacteria) on the (a) likelihood of calling and (b) time spent calling (given that a male produced a call) in *Gryllodes sigillatus*. Points show predicted effects with 95% confidence intervals, taken from MCMCglmm ZAP analysis

The calling effort of 391 males that survived the calling period was measured over two consecutive days following infection cue treatment (sample sizes for diet type and infection cue dose included in appendix Table A1). Diet had a significant effect on time spent calling (see Figure 3 for model coefficients). Males held on the high carbohydrate diet spent more time calling than males maintained on the high protein diet, but the likelihood of calling was the same for both treatments (Figure 4b). Infection cue treatment also influenced male calling effort (Figure 3). We found a significant effect of the low‐dose infection cue (relative to the naive controls) on both the likelihood of calling and the amount of time spent calling (Figure 3). Relative to naive controls, the likelihood of calling for males injected with the low infection cue was lower (Figure 4a), but if they called, they called more (Figure 4b). None of the other infection cue treatments differed from the naive control group with respect to either the likelihood of calling or time spent calling.

### 3.4 Immune measures

Immune measures (circulating hemocytes, presence of microaggregations, antibacterial activity) of 137 males were taken immediately after calling effort trials (sample sizes for diet type and infection cue dose included in appendix Table A1). It should be noted that prior mortality resulting from diet and infection cue treatments limits our interpretation of these data.

**FIGURE A1:**
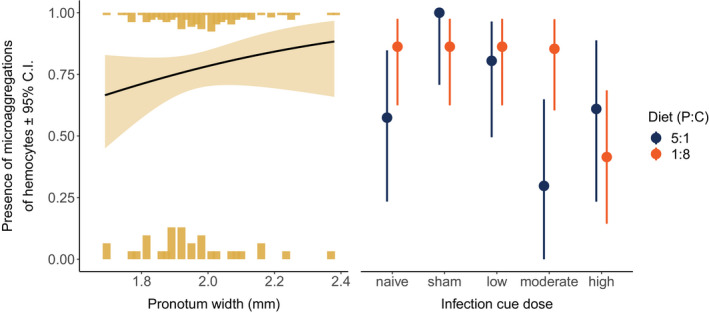
The presence of circulating microaggregations of hemocytes in male *Gryllodes sigillatus* across pronotum width (left) and in response to diet type and infection cue dose (right)

Neither diet type ($\chi_{1,131}^{2}$ = 2.275; *p* = .1315) nor infection cue ($\chi_{4,131}^{2}$ = 2.533; *p* = .6387) significantly influenced the number of circulating hemocytes, after a nonsignificant interaction between diet and infection cue ($\chi_{4,124}^{2}$ = 5.421; *p* = .2468) was removed. However, the presence of circulating microaggregations of hemocytes was significantly influenced by both pronotum width ($\chi_{1,123}^{2}$ = 4.237; *p* = .0396) and the interaction between diet type and infection cue ($\chi_{4,123}^{2}$ = 10.472; *p* = .0332). Larger males were more likely to have circulating microaggregations (see appendix Figure A1). Males fed on the high carbohydrate diet were less likely to have circulating microaggregations if they received the high infection cue dose, and this also tended to be true for males fed the high protein diet and receiving a moderate infection cue dose (Figure A1).

For antibacterial activity of hemolymph, we found a nonsignificant trend for infection cue to affect the zone of growth inhibition of *M. luteus* (*F*_4,125_ = 2.293; *p* = .0631), with antibacterial activity lowest at the high infection cue dose (estimated marginal means ± standard error: naive = 2.248 ± 0.487, sham = 1.457 ± 0.322, low = 1.567 ± 0.326, moderate = 2.189 ± 0.490, high = 0.944 ± 0.218). However, diet type did not significantly influence antibacterial activity (*F*_1,125_ = 0.9194; *p* = .33949; estimated marginal means ± standard error: high protein = 1.66 ± 0.251, high carbohydrate = 1.41 ± 0.172).

### 3.5 Survival after calling trials

Survival of 211 males was tracked daily following the conclusion of calling effort trials (sample sizes for diet type and infection cue dose included in appendix Table A1).

Neither diet type (Cox proportional hazards regression: $\chi_{1}^{2}$ = 0.900; *p* = .3428) nor infection cue dose (Cox proportional hazards regression: $\chi_{4}^{2}$ = 1.371; *p* = .8492) significantly influenced survival following calling effort trials; the nonsignificant interaction between diet and infection cue ($\chi_{4}^{2}$ = 4.598; *p* = .3311) was removed from analysis.

## 4 DISCUSSION

The results of our study show that both macronutrient intake and a simulated infection threat influence the expression of life history traits in decorated crickets, *G. sigillatus*. Specifically, both diet and an immune challenge with heat‐killed bacteria (a) affected male reproductive effort, quantified here as male calling effort, and (b) greatly altered male survival. The two diets used in the current study were deliberately selected because they had been shown previously to either maximize calling effort (P:C = 1:8, high carbohydrate diet) or to maximize immune function, at least with respect to encapsulation ability (P:C = 5:1, high protein diet) (Rapkin et al., 2018), thereby making it more likely that the effects of our immune challenge would be contingent on the diet on which males had been maintained. However, we found no or, at best, weak evidence of an interaction between the diet on which males were maintained and the level of the immune challenge that they experienced in the expression of life history traits. Moreover, although our results aligned with the anticipated effects of these two diets on male calling, surprisingly, they were in apparent contradiction of the anticipated effect on male immunity. We elaborate on these differences below and seek to identify potential mechanisms mediating observed treatment effects and the factors potentially constraining an interaction between diet and the magnitude of an immune challenge on male life history strategy.

Males maintained on a high protein diet exhibited significantly higher mortality than those held on the high carbohydrate diet. Nearly 30% (68 out of 249) of males maintained on the high protein diet died prior to end to the experiment compared with less than 14% (33 out of 245) of males held on the high carbohydrate diet. This is consistent with numerous findings across taxa showing that lifespan is truncated in animals fed a high protein, low carbohydrate diet (reviewed in Le Couteur et al., 2016 and Simpson et al., 2017; Moatt et al., 2020), including *G. sigillatus* (J. Hunt, unpublished data). These recent discoveries, which contrast with conventional Y resource allocation models (van Noordwijk & de Jong, 1986; Zera & Harshman, 2001), have inspired the lethal protein hypothesis (Fanson et al., 2009; Lee et al., 2008; Simpson & Raubenheimer, 2009). Proposed mechanisms mediating the lethal protein hypothesis include increased mitochondrial generation of radical oxygen species (Sanz et al., 2004), changes in the relationship between insulin/IGF‐1 and amino acid signaling (e.g., TOR) pathways (Kapahi et al., 2004), and damage to organs from nitrogenous waste products. Further studies are needed to conclusively determine whether the diet‐induced mortality in the present study is due to an excess of protein or a deficit of carbohydrates. Males were always provided more food than they could eat, suggesting that they were not carbohydrate limited, and males held on the high protein diet consumed a greater amount of food than those held on the high carbohydrate diet across the entire range of body sizes, which could have been due to a greater nutritional demand for carbohydrates in the former. Indeed, Rapkin et al. (2018) showed that when male *G. sigillatus* are given a dietary choice, they regulate their intake of protein and carbohydrate to a P:C ratio of 1:2. Still, providing these diets ad libitum does not rule out the possibility that males fed our high protein diet were carbohydrate limited. For example, there are likely size constraints on the amount of food individuals can consume at a time; perhaps larger males were able to eat more food simply because of a greater gut capacity. Moreover, it may be that consuming a particular target amount of specific nutrients triggers feeding cessation, which could result in a deficiency of less abundant nutrients. For example, if male *G. sigillatus* feed until they reach some level of protein intake (e.g., to limit the aforementioned negative effects of excess protein), those that were fed on our high protein diet may have become carbohydrate limited over time.

As was the case with diet, infection cue dose had a significant effect on male survival through the end of the 2‐day calling period. Specifically, the highest infection cue dose resulted in higher male mortality than any of the other infection cue levels. This suggests that our simulated infection threat imposed a physiological cost on males and that at the most severe infection threat level, this cost was manifest in increased mortality. This is consistent with a cost of using the immune system (Sadd & Schmid‐Hempel, 2009), which has been shown to have consequences for survival in other insect taxa (Armitage et al., 2003; Moret & Schmid‐Hempel, 2000). This has also previously been shown to be independent of nutritional condition in another cricket species (Jacot et al., 2004).

Both diet and infection cue treatment had significant effects on male reproductive effort, measured here as the time spent calling to attract a mate (termed calling effort). Calling effort was significantly higher in males held on the high carbohydrate diet than in those held on the high protein diet, consistent with previous work (Rapkin et al., 2018). We also found that infection cue, specifically at a low dose, impacts male reproductive effort. If they called, males injected with a low infection cue dose called more relative to naive controls. This increased calling effort is consistent with a pattern of terminal investment (Duffield et al., 2017), but, surprisingly, neither of the two higher infection cue treatments differed from the naive control group with respect to calling effort. However, at least in the case of males injected with the high infection cue dose, males experienced at least a twofold higher mortality during the calling period compared with males in the other infection cue treatments, and thus, males in this group were, to some extent, self‐selected. If the males that died were those that were more inclined to terminally invest, whereas those that survived prioritized maintenance over reproduction, then this would attenuate any effect of infection cue treatment on calling effort at the more severe infection threat levels.

Although diet and infection cue treatment independently influenced male calling effort, we found no evidence of an interaction between these two factors. The absence of such an interaction contrasts with an early study demonstrating an interaction between male age and infection cue dose, in which older male *G. sigillatus* increased their calling effort in response to the same graded increase in infection threat used here, whereas younger males did not (Duffield et al., 2018). Why do males retain this level of plasticity in calling effort with respect to age, but not diet? One possibility is that age overrides any influence of diet on the propensity to shift life history strategy, given that it has been previously shown that males at the age used in this study exhibit terminal investment, whereas younger males do not (Duffield et al., 2018). A standard laboratory diet was provided in this previous study, but it is possible that terminal investment in younger males could be elicited by certain combinations of diet and infection cue dose, in line with the dynamic terminal investment threshold model (Duffield et al., 2017). An alternative is that males were terminally investing in other important components of reproductive effort that we did not measure in the present study. For example, the spermatophore transferred by males at copulation includes a large gelatinous mass, the spermatophylax, that the female consumes after mating as a nuptial food gift, which is critical to male fertilization success (Eggert et al., 2003; Sakaluk, 1984, 1986; Sakaluk & Eggert, 1996). Duffield et al. (2015) showed that immune‐challenged males terminally invest by altering the free amino acid profile of the spermatophylax, enhancing its gustatory appeal to females (see also Gershman et al., 2012). Moreover, male chemical cues, in the form of cuticular hydrocarbons (CHCs), greatly influence whether a female mounts a male, a necessary antecedent to copulation (Capodeanu‐Nägler et al., 2014; Weddle et al., 2013). The CHC profile of a male, and by extension, his attractiveness, can be significantly influenced by his nutritional environment (Weddle et al., 2012). Both CHC expression (Rapkin et al., 2016) and the amino acid composition of the spermatophylax (Rapkin, et al., 2017) are optimized at a P:C ratio of 1:1.3 and 1:1.5, respectively, ratios far removed from the two diets offered in the present study.

Despite our initial expectation, we found no evidence that males held on the high protein diet exhibit enhanced immune function. Specifically, there was no positive effect of diet on the number of circulating hemocytes, presence of microaggregations, or antibacterial activity of the hemolymph. We did, however, find a significant interaction between diet and infection cue dose on the incidence of circulating microaggregations of hemocytes, suggesting that both factors could affect this immune response. This contrasts with the results of a previous study showing that among 24 diets differing in P∶C ratio and distributed along six nutritional rails, a P:C = 5:1 ratio optimizes immune function as assessed using an encapsulation assay (Rapkin et al., 2018). In the present study, however, we did not measure the encapsulation ability of males, and the difference between the two studies may be a function of the different components of immunity that were measured. Different facets of immunity can be triggered by different types of threats or regulated independently, resulting in positive, negative, or no associations between immune components (Adamo, 2004; Forsman et al., 2008). Indeed, although Rapkin et al. (2018) demonstrated a significant effect of macronutrient intake on male encapsulation ability, they found no effect of diet on the activity of phenoloxidase, an important enzyme in the melanization cascade. Ultimately, we are cautious to draw any firm conclusions about the influence of diet and infection cue on immune parameters, due to the interesting but confounding differential effects of these factors on survival prior to the assaying of immunity.

In conclusion, both macronutrient intake and a simulated infection threat independently influenced the survival and reproductive effort of male *G. sigillatus*. There was evidence for terminal investment, as males increased calling effort at the low infection cue dose, but interpretation of responses at the higher threat levels was hampered by the differential mortality of males across diet and infection cue treatments, the latter demonstrating a cost of immune activation for survival. There was, however, no evidence of an interaction between diet and infection cue dose in their influence on calling effort, suggesting that the threshold for terminal investment was not contingent on diet, in contrast to earlier work documenting a shifting terminal investment threshold contingent on male age (Duffield et al., 2018). The absence of a dynamic terminal investment threshold may have been due to the previously documented influence of age masking other intrinsic and extrinsic factors, or, alternatively, males prioritizing investment in other components of reproductive effort. Regardless, how reproductive effort changes in accordance with the various intrinsic and extrinsic factors that alter an individual's residual reproductive value remains a fertile area of inquiry (Duffield et al., 2017), but any generalizations must await additional comparative studies that preferably incorporate multiple facets of male reproductive effort.

## CONFLICT OF INTEREST

No competing interests declared.

## AUTHOR CONTRIBUTIONS

**Kristin R. Duffield:** Conceptualization (lead); data curation (lead); formal analysis (lead); funding acquisition (supporting); investigation (lead); methodology (lead); visualization (lead); writing – original draft (lead); writing – review & editing (equal). **Kylie J. Hampton:** Investigation (supporting); writing – review & editing (equal). **Thomas M. Houslay:** Formal analysis (supporting); methodology (supporting); writing – review & editing (equal). **James Rapkin:** Methodology (supporting); resources (equal); writing – review & editing (equal). **John Hunt:** Funding acquisition (supporting); methodology (supporting); resources (equal); writing – review & editing (equal). **Ben M. Sadd:** Conceptualization (supporting); funding acquisition (equal); methodology (equal); resources (equal); supervision (lead); writing – original draft (supporting); writing – review & editing (equal). **Scott K. Sakaluk:** Conceptualization (supporting); funding acquisition (equal); methodology (supporting); resources (equal); supervision (lead); writing – original draft (supporting); writing – review & editing (equal).

## EEO/NON‐DISCRIMINATION STATEMENT

USDA is an equal opportunity provider and employer.

## Data Availability

Data deposited at Dryad: https://doi.org/10.5061/dryad.3ffbg79gc.

## Appendix A

**TABLE A1:** Sample sizes for measurements of calling effort, survival post‐calling effort trials, and immune function (circulating hemocytes, presence of microaggregations, antibacterial activity) of male *Gryllodes sigillatus* fed either of two diet types and administered a treatment from an increasing spectrum of infection cue intensity

Diet	Infection cue	Calling	Survival	Immune function
P:C = 5:1	Naive	33	20	11
	Sham	36	21	11
	Low	35	21	12
	Moderate	34	20	11
	High	41	14	9
P:C = 1:8	Naive	40	23	17
	Sham	41	23	17
	Low	41	24	17
	Moderate	43	25	16
	High	47	20	16
Total		391	211	137
